# Antimicrobial Resistance Profile of Bacterial Isolates Identified from Surgical Site Infections at a Referral Hospital, Northwest Ethiopia

**DOI:** 10.4314/ejhs.v31i3.21

**Published:** 2021-05

**Authors:** Samuel Abosse, Chalachew Genet, Awoke Derbie

**Affiliations:** 1 College of Health Sciences, Asossa University, Ethiopia; 2 Department of Medical Microbiology, College of Medicine and Health Sciences, Bahir Dar University, Ethiopia; 3 Centre for Innovative Drug Development and Therapeutic Trials for Africa (CDT-Africa), Addis Ababa University, Ethiopia; 4 Department of Health Biotechnology, Biotechnology Research Institute, Bahir Dar University, Ethiopia

**Keywords:** Surgical wound infection, antimicrobial resistance, Felege Hiwot Referral Hospital

## Abstract

**Background:**

Surgical wound infections (SWI) remain as a major source of postoperative illness that increases the time of hospital stay and health care related costs globally. On top of this, the emergence and spread of drug-resistant pathogens continue to challenge the proper management of surgical wound infections.

**Methods:**

A hospital based cross-sectional study was conducted at Felege Hiwot Referral Hospital (FHRH). A total of 165 study participants were included. Socio-demographic data were collected using a pre-tested structured questionnaire. Isolates were identified by conventional bacteriological technique and antimicrobial susceptibility test was performed using the Kirby-Bauer disc diffusion method.

**Results:**

Among 165 study participants, 98 (59.4%) were males. The overall prevalence of culture confirmed surgical wound infection was 115 (69.7%). A total of 125 bacteria isolates were identified among which, Staphylococcus aureus was predominant followed by Pseudomonas aeruginosa and Klebsiella species with a proportion of 31 (24.8%), 26 (20.8%) and 17 (13.6%), respectively. Majority (80.8%) of the isolates were found multidrug resistant (MDR). Dirty wound and duration of hospital stay were found significantly associated with culture confirmed surgical wound infections.

**Conclusions:**

S. aureus, P. aeruginosa and Klebsiella species were the most common isolates identified from surgical wound sites. Most of these pathogens were found MDR. Therefore, regular surveillance on the types of bacterial isolates and their drug resistance pattern should be considered.

## Introduction

Surgical wound infection (SWI) develops at or near a surgical incision within 30 days of the procedure or within 1 year if the procedure includes implanted device or prosthesis (1). It is the second common healthcare associated infections in hospitalized patients, after urinary tract infections, and cause different complications that increase morbidity, mortality, duration of stay in hospital and health care cost (2, 3). Different microorganisms including bacteria, fungi and viruses can cause SWI.

The common bacterial pathogens associated with SWIs include, *Staphylococcus aureus*, *Pseudomonas aeruginosa, Escherichia coli, Klebsiella spp., Enterobacter* spp.*, Proteus* spp.*, Acinetobacter* spp. and *Serratia* spp (3, 5).

The sources of bacteria for SWIs can be patient's own normal flora, the hospital environment, hospital staffs and visitors. The type of the causative microorganisms may vary from hospital to hospital and development of SWIs is highly depends on patient factors, wound type and drugs prescribed to the patients (6, 7, 8).

The diagnosis and management of SWI in Ethiopia is most often relied on the medical history of individuals (9, 12). However, the clinical characteristics of SWI cannot be consistently used to establish the etiologic diagnosis with adequate sensitivity and specificity. The prevalence of culture confirmed SWI in Ethiopia varies in different geographical area ranging from 3.5% to 84.1% (5, 9–13).

An ever-increasing pattern of antimicrobial resistance by commonly identified bacterial pathogenes from SWIs is a major public health problem making SWIs management difficult. The problem is more pronounced in resource limited countries where rational use of antibiotics is highly compromised (8, 7, 14). In Ethiopia, treatment of SWI is made usually empirically, in which the etiologic agent is rarely identified. Therefore, identifying the most common bacterial pathogens associated with SWI and their drug resistance profile would be valuable for good clinical practice and to reduce morbidity and mortality associated with it.

There is limited data about the bacterial and antimicrobial resistance profile SWI in the present study area. Therefore, this study was conducted to provide data on the type of bacterial pathogens and their drug resistance profile among SWI patients at Felege Hiwot Referral Hospital (FHRH), Bahir Dar, Northwest Ethiopia.

## Methods

**Study design, period and setting:** Hospital based cross sectional study was conducted from 1 February to 30 June, 2019 at Felege Hiwot Referral Hospital. The hospital was established in 1952 and has been providing service for people living in Bahir Dar and the surrounding Woredas and kebeles. The hospital has 13 wards including surgical ward (SW), orthopedic ward (OPW) and medical ward (MW). There were 430 beds and about 531 health professionals with close to 600 average daily clients visiting the hospital routinely during the study period. The hospital also serves as a practical attachment site for different health science students coming from non-governmental and government higher institutes including Bahir Dar University.

**Study population:** All patients presumptive for surgical wound infections at Felege Hiwot Referral Hospital were considered as a source population. Likewise, patients with clinically diagnosed for surgical wound infection and who were available in surgical ward, outpatient department and medical ward during the study period were considered as the study population.

All patients clinically diagnosed for SWI during the study period in SW, OPW and MW and gave written informed consent/assent to participate in the study were included. Patients clinically diagnosed as having SWI in SW, OPW & MW but unconscious during the study period were excluded.

**Sampling and data collection:** A total of 165 study participants (99, 47 and 19 patients admitted in SW, OPW and MW respectively) were included using a single population proportion formula taking 10.9% SWI prevalence from previous studies in northwest Ethiopia (9), 5% margin of error and 95% level of confidence. A convenient sampling method was used to select study participants until required number was achieved.

A pre-tested questionnaire, which developed after consulting previous publications (5, 9, 10, 11, 12, 13), was used to collect socio-demographic and clinical data using face-to-face interview. For bacteriological culture and antimicrobial susceptibility testing (AST), wound secretion/pus samples were collected using sterile cotton swabs following the standard bacteriological procedure. The collected samples were immediately immersed in test tube containing Brain Heart Infusion (BHI) transport medium and transported to FHRH microbiology laboratory for processing (9, 15, 16). The swab was inoculated onto 5% Blood agar ((Oxoid, Ltd., UK) plate (BAP), MacConkey agar plat (Oxoid, Ltd., UK) and Mannitol salt agar plat (Oxoid, Ltd., UK) aseptically. The inoculated plates were incubated aerobically for 24 hours at 37°C and bacterial identification was done using the standard bacteriological technique (16, 17, 18).

Antimicrobial susceptibility testing was done by the Kirby-Bauer disc diffusion method on Mueller Hinton agar plate (Oxoid Ltd., UK). The inoculums were prepared from pure culture by picking parts of similar test bacteria with a sterile wire-loop and were suspended in sterile normal saline. The inoculum turbidity was adjusted to 0.5 McFarland standards. The bacterial isolates were tested using the following antibiotics (Oxoid Ltd., UK): Oxacillin (1µg), Chloramphenicol (30µg), Ciprofloxacin (5µg), Clindamycin (2µg), Tetracycline (30µg), Erythromycin (15µg) and Cefoxitin (30µg), Gentamycin (10µg), Amoxicillin (25µg), Augmentin (20/10µg), Ceftazidime(30µg), meropenem (MEM, 10µg), and cefotaxime (CTX, 30µg) which were selected based on the CLSI guideline (19) and local prescription protocol. The AST result was interpreted based on clinical laboratory standards institute (CLSI) guideline (19).

Moreover, *Staphylococcus aureus* (ATCC 25923), *Escherichia coli* (ATCC-25922) and *Pseudomonas aeruginosa* (ATCC-27853) were used as a quality control strains for culture and antimicrobial susceptibility testing (19).

**Data analysis:** The collected data were entered and analyzed using SPSS version 23. Descriptive statistics was used to describe the sociodemographic & clinical characteristics of the study participants, bacterial isolates and their AMR profile. Logistic regression was run to find factors associated with culture confirmed SWIs and *p*-value <0.05 was considered as statistically significant.

**Ethical Approval**: Ethical clearance was obtained from Institutional Review Board of Bahir Dar University. All patient data were kept confidential.

## Results

**Socio-demographic and clinical characteristics of study participants:** Among the 165 study participants, 98 (59.4%) and 87 (52.7%) were males and within the age groups of 16–40 years, respectively. About 58.2% of the study participants were from rural setting. Further, the most common type of SWI at 40.6% was on the abdomen (Table-1).

**Table 1:** Socio-demographic and clinical characteristics of study participants presumptive for SWI at FHRH, February 2019

Variable	Frequency	Percent
**Sex**		
**Male**	98	59.4
**Female**	67	40.6
**Age**		
**≤15**	37	22.4
**16–40**	87	52.7
**41–60**	31	18.8
**≥61**	10	6.1
**Education level**		
**No education**	42	25.5
**1–8 school**	79	47.9
**9–12 school**	23	13.9
**College and above**	21	12.7
**Occupation**		
**Students**	52	31.5
**Farmers**	59	35.7
**Government** **employee**	18	10.9
**Merchant**	25	15.2
**Privates employee**	11	6.7
**Residence**		
**Urban**	69	41.8
**Rural**	96	58.2
**Site of SWI**		
**Abdomen**	67	40.6
**Leg or lower part**	41	24.8
**Shoulder or upper** **part**	26	15.8
**Back bone**	17	10.3
**Face, head and neck**	14	8.5
**Wound type**		
**Clean**	30	18.2
**Clean-contaminated**	51	30.9
**Contaminated**	43	26.1
**Dirty**	41	24.8
**Duration of stay in** **hospital**		
**≤ one week**	56	33.9
**1–2 weeks**	68	41.2
**2–3 weeks**	28	17.0
**3–4 weeks**	13	7.9

**Profile of the identified isolates:** The overall prevalence of culture confirmed SWI was 115 (69.7%) where 91.3% were infected by a single bacterial type. A total of 125 bacteria were identified of which 94 (75.2%) were Gram negative. The most predominant isolate was *S. aureus* followed by *P. aeruginosa* and *Klebsiella* spp. at a frequency of 31(24.8%), 26(20.8%) and 17(13.6%), respectively. Moreover, the proportion of *S. aureus* & *P. aeruginosa* co-growth was at 50% (Table 2).

**Table-2:** Bacterial profile of SWI patients attending at FHRH, February 2019

SWI cases (n=115)	Frequency	Percentage
**Single Gram positive isolates (n=24)**		
***S. aureus***	24	20.9
**Single Gram negative isolates (n=81)**		
***P. aeruginosa***	19	16.5
***Klebsiella* spp.**	16	13.9
***Citrobacter* spp.**	10	8.7
***E. coli***	12	10.4
***A. baumannii***	8	7
***Proteus* spp.**	7	6.1
***Enterobacter* spp.**	6	5.2
***Serratia* spp.**	3	2.6
**Mixed Gram-negative isolates (n=3)**		
***P. aeruginosa* & *Klebsiella* spp.**	1	0.87
***P. aeruginosa* & *A. baumannii***	1	0.87
***Citrobacter* spp. & *Enterobacter* spp.**	1	0.87
**Mixed Gram positive & negative isolates (n=7)**		
***S. aureus* & *P. aeruginosa***	5	4.35
***S. aureus* & *Proteus* spp.**	2	1.74

**Total**	**115**	**100**

SWI: Surgical wound infection, Single isolate= when one bacterial species/genus identified, Mixed isolates= when two bacterial species/genus identified

**Antimicrobial resistance profile of the isolates:***S. aureus* showed high level of resistance to amoxicillin (87.1%) and ampicillin (71%). On the other hand, it showed low level of resistance to vancomycin and ciprofloxacin with a proportion of 9.7% and 16.1%, respectively (Table 3).

**Table 3:** Antimicrobial resistance profile of S. aureus isolated from patients with SWI at FHRH, February 2019

Bacterial isolates	Resistant isolate: Number (%)									
	
	Amo	Amp	Ox	Ery	Gen	Cip	Ch	Tet	Van	Cl
***S. aureus***	27	22	10	12	5	5	11	12	3	8
**(n-31)**	(87.1)	(71.0)	(32.3)	(38.7)	(16.1)	(16.1)	(35.5)	(38.7)	(9.7)	(25.8)

**Note:** Amo: amoxicillin, Amp: Ampicillin, Ox: Oxacillin, Ery: Erythromycin, Gen:Gentamycin, Cip: Ciprofloxacin, Ch: Chloramphenicol, Tet: Tetracycline, Van:Vancomycin, Cl: Clindamycin

The predominant Gram-negative isolates *P. aeruginosa* and *Klebsiella* spp. showed 84.6% and 82.4% resistance to ampicillin, respectively. Furthermore, all Gram-negative isolates exhibited more than 81% resistance to ampicillin. On the other hand, *P. aeruginosa* showed low-level resistance to ciprofloxacin (19.2%) and meropenem (23.1%). Similarly, *Klebsiella* spp. showed low-level resistance to meropenem (11.8%) and gentamycin (11.8%) (Table 4)

**Table 4:** Antimicrobial resistance profile of Gram-negative bacteria isolated from patients with SWI at FHRH, February 2019

Bacterial isolates (n=94)	Resistant isolate: Number (%)									

	Amp	0Mem	Gen	Cip	Ch	Tet	Ag	Ctx	Cef	Cez
***P. aeruginosa*** **(n=26)**	22 (84.6)	6 (23.1)	10 (38.5)	5 (19.2)	9 (34.6)	13 (50.0)	14 (53.9)	16 (61.5)	15 (57.7)	14 (53.9)
***Klebsiella* Spp.** **(n=17)**	14 (82.4)	2 (11.8)	2 (11.8)	4 (23.5)	5 (29.4)	10 (58.8)	8 (47.1)	9 (52.9)	10 (58.8)	9 (52.9)
***E. coli* (n=12)**	10 (83.3)	2 (16.7)	4 (33.3)	5 (41.7)	5 (41.7)	7 (58.3)	7 (58.3)	8 (66.7)	7 (58.3)	9 (75.0)
***Citrobacter*** **Spp. (n=11)**	9 (81.8)	5 (45.5)	4 (36.4)	5 (45.5)	3 (27.3)	8 (72.7)	5 (45.5)	9 (81.8)	8 (72.7)	8 (72.7)
***Acinetobacter*** **Spp. (n=9)**	8 (88.9)	3 (33.3)	2 (22.2)	3 (33.3)	4 (44.4)	7 (77.8)	6 (66.7)	7 (77.8)	8 (88.9)	6 (66.7)
***Proteus* Spp.** **(n=9)**	8 (88.9)	2 (22.2)	2 (22.2)	4 (44.4)	4 (44.4)	7 (77.8)	5 (55.6)	6 (66.7)	7 (77.8)	6 (66.7)
***Enterobacter*** **Spp. (n=7)**	6 (85.7)	1 (14.3)	2 (28.6)	3 (42.9)	3 (42.9)	4 (57.1)	3 (42.9)	5 (71.4)	4 (57.1)	5 (71.4)
***Serratia* Spp.** **(n=3)**	3 (100)	0 (0)	0 (0)	1 (33.3)	2 (66.7)	2 (66.7)	1 (33.3)	2 (66.7)	1 (33.3)	1 (66.7)

**Note:** Amp: Ampicillin, Mem: Meropenem, Gen: Gentamycin, Cip: Ciprofloxacin, Ch:Chloramphenicol, Tet: Tetracycline, Ag: Augmentin, Ctx: cefotaxime, Cef: Cefoxitin, Cez: Ceftazidime

**Figure 1:**
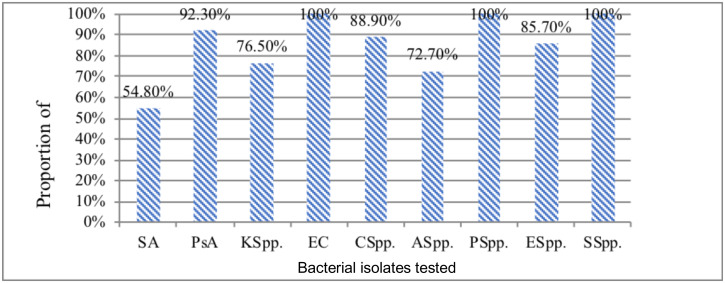
Multidrug resistance profile of bacterial isolates from patients with SWI at FHRH, Feb 2019. **Note**: SA: *S. aureus*, PsA: *P. aeruginosa*, KSpp: *Klebsiella* Spp., EC: *E. coli*, CSpp: *Citrobacter* Spp., ASpp: *Acinetobacter* Spp., PSpp: *Proteus* Spp., ESpp: *Enterobacter* Spp., SSpp: *Serratia* Spp., MDR: Multidrug resistance

The overall multi-drug resistance (MDR) level was at 80.8%. MDR was defined as the ability of an isolate to resist >3 antimicrobial agents in different class. Relatively Gram negatives showed higher MDR level (89.4%) than Gram positives (54.8%). Moreover *E. coli, Proteus* spp. and *Serratia* spp. isolates exhibited 100% level of MDR (Figure 1).

**Factors associated with culture positive surgical wound infection:** Patients who had dirty wound were more than 36 times more likely to develop culture confirmed SWIs than those having clean wound (AOR: 36.406; CI: 7.115–185.281). Moreover, a surgical wound with more than four weeks of duration was found more than 13 times more likely to be positive for bacteriological culture (AOR: 13.75; 95%CI: 1.7–114.13 (Table 5). Other variables like participants' age, sex and surgical site didn't show statistical significant association.

**Table 5:** Factors associated with culture positive SWI at FHRH, February 1 to June 30, 2019

Variables	Bacterial occurrence		COR, 95% CI	(AOR;95% CI), *p*-value

	Positive: N (%)	Negative: N (%)		
**Wound type**				
Clean	11 (9.65%)	19(37.25%)	1	1
Clean-contaminate	31 (27.19%)	20(39.23%)	78.2, 23.5–26.8	(2; 0.739–5.452), 0.172
Contaminate	33 (28.95%)	10(19.61%)	10.4, 3.1–35.3	(5.5; 1.891–16.097), 0.002
Dirty	39 (34.21%)	2 (3.92%)	18.4, 2.0–176.4	(36.4; 7.115–185.281), 0.001
Duration of hospital stay				
**≤1 week**	24 (21.05%)	30(58.82%)	1	1
**1–2week**	59 (51.75%)	14(27.45%)	4.1, 1.9–9.1	(5.3; 2.385–11.633), 0.001
**2–3week**	20 (17.54%0	6 (11.76%)	3.2, 1.2–8.2	(4.2; 1.446–12.009), 0.008
**≥4 week**	11 (9.65%)	1 (1.96%)	12.9, 1.6–105.9	(13.8; 1.657–114.132), 0.015

CI: confidence interval; AOR: adjusted odds ratio

## Discussion

The emergence and spread of antimicrobial resistant pathogens is associated with serious public health outcomes (19). Patients with SWI caused by antibiotic resistant pathogens are at increased risk of worse clinical outcomes and consume more health-care resources compared with their counterparts.

In the present study, the proportion of culture confirmed SWI was 69.7%. This finding was similar with other studies done in Ethiopia and abroad; Mekelle (5), Hawassa (8) and Nepal (20) that reported 75%, 71.1% and 64.5% culture positive surgical wound, respectively. On the other hand, our finding was higher than a report from Gondar (3.5%) (13) and Bahir Dar (10.9%) [9]. In contrast, it was found much lower than other studies done in Ethiopia (10, 12, 21) and somewhere (22, 23) with a proportion of 84.1–92% and 77.6–100%, respectively. The difference in the proportion of culture confirmed SWI might be due to differences in the distribution of nosocomial pathogens and infection prevention & control practices among different countries and health facilities.

In our study, the predominant isolate was *S. aureus* (24. 8%). This finding was in line with previous reports in Ethiopia that reported 23.4–35.8% proportion (5, 9, 13, 21). The dominance of *S. aureus* from would swab might be because the bacterium can be found colonizing fomites and health professionals that can act as a source of SWIs. *P. aeruginosa, Klebsiella* species and *E. coli* were the common isolate we have reported next to *S. aureus* with a proportion of 20.8%, 13.6% and 9.6, respectively. Comparable figures were reported previously in Ethiopia (5, 9, 21). This amount of gram-negative isolates in the present study might be because of the contamination of the surgical wounds with gastrointestinal tract flora as majority of the surgery in the present study were abdominal type that accounted about 40.6%. Additionally, the hospital environment and the surgical equipment might serve as a source of infection by these type of isolates (24–29).

Bacterial isolates in the present study showed different antimicrobial sensitivity profile to the various antibiotics they were tested. The predominant isolate, *S. aureus,* showed higher level of resistance to amoxicillin and ampicillin at 87.1% & 71.0%, respectively. On the other hand, it showed the least resistance to vancomycin (9.7%) followed by ciprofloxacin (16.1%) and gentamycin (16.1%). This finding was comparable with a similar study done in Hawassa (21) that reported 100% and 20% resistance of *S. aureus* to amoxicillin and gentamycin, respectively. Likewise, similar finding was also reported in Mekelle (5). Gramnegative isolates *P. aeruginosa, Klebsiella spp.*, & *E. coli* showed more than 82%, 50%, 47% and 11% level of resistance to ampicillin, tetracycline, augmentin and gentamycin, respectively. A comparable result on the resistance of ampicillin (89.7%), tetracycline (93.1%) and gentamycin (27.8%) was reported in Mekelle (5) and Hawassa (8). Moreover, these isolates showed more than 29% and 19% level of resistance to chloramphenicol and ciprofloxacin, respectively. This finding was in line with a study done in Nigeria (23) that reported >38% and 15% resistance to chloramphenicol and ciprofloxacin, respectively.

In our study the overall proportion of multi-drug resistance (MDR) was at 80.8% which is consistent with similar study findings in Ethiopia that reported 82.9–84.7% MDR level among isolates from surgical wound swab (5, 8). Equally, a study from Nepal reported 89.5% level of MDR (20). In contrast, other studies in Nepal and Uganda reported higher MDR proportion among isolates from surgical wound (20, 22, 28). The reported an increased MDR might be attributed by a number of factors including, over and misuse of antimicrobials in the study area where there is weak regulatory practice and inadequate bacteriological surveillance due to lack of routine antimicrobial susceptibility testing facilities. Most of the antimicrobials listed are freely available in local pharmacies and people could purchase and use them without prescription. This would also play its share for an increased antimicrobial resistance in this study.

In the present study, wound type and duration of hospital stay were significantly associated with culture confirmed SWIs. These factors were also reported by other similar studies done in Ethiopia (5, 9). A study by Fisha and his colleague supported our finding in which dirty wounds and length of hospital stay were found predictors of surgical site infections among patients in public hospitals of Ethiopia (30).

Our study provided important data on the type of bacterial isolates, their AMR profile and factors associated with SWI in the study area. However, the study has some confines that should be considered while interpreting the finding. No attempt was made to isolate anaerobic bacterial pathogens that could be associated with SWIs. Moreover, species level identification of certain bacterial genus was not done due to resource limitation

In conclusion, in our study *S. aureus* was the predominant isolate followed by *P. aeruginosa* and *Klebsiella* species from surgical wound. Besides, these isolates were found resistant to commonly prescribed antimicrobials. Additionally, >80% of the isolates were found MDR. Type of wound and longer duration of hospital stay were found significantly associated with SWI. Therefore, proper SWI prevention measures should be in place. Further, actions to reduce antimicrobial resistance should be strengthened. Rational use of antimicrobials, collaborative regular surveillance of pathogens associated with SWI with their antimicrobial resistance pattern should be considered in the study area.
